# Participation in cancer survivorship survey research: Differences by rurality and age

**DOI:** 10.1017/cts.2025.10123

**Published:** 2025-09-10

**Authors:** Emily Hallgren, Aaron R. Caldwell, Jennifer A. Andersen, Mohammed Ason, James P. Selig, Jonathan Langner, Pearl A. McElfish

**Affiliations:** 1 Larner College of Medicine, University of Vermont, Burlington, VT, USA; 2 University of Vermont Cancer Center, Burlington, VT, USA; 3 College of Medicine, University of Arkansas for Medical Sciences Northwest, Springdale, AR, USA; 4 Institute for Community Health Innovation, University of Arkansas for Medical Sciences Northwest, Springdale, AR, USA; 5 Fay W. Boozman College of Public Health, University of Arkansas for Medical Sciences Northwest, Springdale, AR, USA

**Keywords:** Cancer survivors, cancer survivorship research, survey participation, rurality, age

## Abstract

**Introduction:**

Rural cancer survivors have worse outcomes than their urban counterparts. To improve outcomes, it is essential that rural survivors participate in research, yet they are underrepresented in cancer research. The aim of this study was to assess urban-rural differences in participation in a cancer survivorship survey and differences in mode of participation (mail, online, or phone) by rurality and age.

**Methods:**

We developed a survivorship needs assessment survey and invited cancer survivors to participate by mail, online, or phone. We compared participation between rural and urban invitees and examined differences in mode of participation by rurality and age.

**Results:**

A quarter (25.47%) of invited rural patients and 27.84% of invited urban patients participated in the survivorship study. The probability of participation by urban survivors was approximately 1.09 times higher than for rural survivors (*χ* ^2^(1) = 4.31, *p* = 0.038). Rural survivors were more likely to participate by mail (average difference [Rural-Urban] = 9.64%, *p* < 0.001), while urban survivors were more likely to participate online (average difference [Urban-Rural] = 8.77%, *p* < 0.001). As participant age increased, the likelihood of survey participation by mail increased (1.16% per year of age, *p* < 0.001) while the probability of participating online decreased by 1.20% per year of age (*p* < 0.001).

**Conclusion:**

To ensure equitable access to research for rural and older cancer survivors, researchers should design studies with a range of participation modes. Non-digital methods, such as mailed paper surveys, appear to promote participation among rural and older survivors.

## Introduction

Rural cancer patients and survivors suffer worse outcomes than their urban counterparts, including poorer treatment outcomes, higher mortality, worse physical and mental health, and lower quality of life [1–4]. To improve cancer outcomes among rural residents, we must identify the factors driving rural cancer disparities. To do so, it is essential that rural cancer patients and survivors participate in cancer population science research.

Rural residents, however, are underrepresented in health research broadly [5,6] and cancer survivorship research specifically [7–9]. Rural populations have not been prioritized in cancer survivorship research until recently [9,10] and have been largely excluded from important bodies of work, including geriatric oncology [7]. Gellar et al. (2011) found rural residence was associated with a lower likelihood of participating in a Survivor Registry in Vermont, with 35.1% of invited urban cancer survivors agreeing to participate in the registry compared to 31% of invited rural cancer survivors.

However, extant research suggests rural residents are as interested in health and cancer research as urban residents[11–13]. In the state of Arkansas, McElfish and colleagues (2018) found rural residents were as willing to participate in health research as urban residents. Caston (2022) found similar rates of interest in cancer clinical trials among rural and urban patients with cancer. Similarly, a scoping review by McPhee et al. (2022) found that rural residents are interested in cancer clinical trials and, in some studies, participated at higher rates than the United States (U.S.) national average, but face barriers to participation. This prior research suggests the issue is not a lack of interest on the part of rural populations, but is rather barriers to participation (e.g., limited access to broadband internet) and lack of effort to ensure the inclusion of rural communities in research studies.

Rural residents in the U.S. are also older than urban residents overall [14], and research suggests older cancer survivors are less likely to participate in research [8]. Gellar et al. (2011) found that younger cancer survivors were more likely to participate in a survivor registry, with participation declining with age. Some literature indicates older cancer survivors are not invited to participate in cancer research at the same rates as younger survivors [15].

In a 2020 policy statement on Cancer Disparities and Health Equity, the American Society of Clinical Oncology (ASCO) included “Ensure Equitable Access to Research” as a key recommendation to promote cancer health equity [16]. The policy statement noted that researchers should be encouraged to employ recruitment strategies that ensure adequate representation of social groups disproportionately affected by cancer and at risk of disparate outcomes, including rural populations [16]. However, the identification of participation methods that are most effective for rural and other underrepresented populations remains under-explored in the literature. The overall goal of this study was to elucidate whether certain data collection modes promote participation among rural and older cancer survivors, two groups who are underrepresented in cancer research.

The aims of this study were, first, to assess whether rural or urban cancer survivors were more likely to participate in a survivorship survey; and second, to assess whether rural and urban cancer survivors were more likely to participate in the survivorship survey via different modes (i.e., by mail, online, or phone). Because rural residents are generally older than urban residents, we also sought to examine whether the mode of survey participation differed by age and if there was a combined effect of rurality and age on the mode of survivorship survey participation.

## Materials and methods

### Study overview

This study was approved by the Institutional Review Board (protocol #274075) at the University of Arkansas for Medical Sciences (UAMS). The *Cancer Survivor-Caregiver Assessment* was a needs assessment of cancer survivors who received a cancer diagnosis and/or cancer treatment at UAMS within the past three years (excluding the most recent six months to reduce patient burden for those newly diagnosed). In addition to the cancer survivor, the survivor’s caregivers were also invited to participate in a separate survey. The goal of the *Cancer Survivor-Caregiver Assessment* was to examine the health, quality of life, and unmet needs of cancer survivors and their caregivers.

### Recruitment

Patients’ contact information was abstracted from the electronic medical record (EMR), and they were recruited by mail, e-mail, and/or text message. First, we sent a mailed packet to all invited participants containing a study flyer, a study letter describing the study and inviting them to participate, a paper copy of the survey, a pre-paid return envelope, and a small incentive (a magnet calendar). The study letter included signatures of the principal investigator, several UAMS oncology physicians, and the name of an oncology social work department program manager with whom patients frequently interact for patient services.

For those who had e-mail addresses listed in their medical records, we then sent an e-mailed invitation containing the study flyer, study letter, and a link to complete the survey online via REDCap. Patients received up to three reminder e-mails depending on whether and when they completed the survey. For those who had cell phone numbers listed in their medical record, we sent a text message with the study flyer, study letter, and a link to complete the survey online via REDCap. Patients received up to three reminder text messages depending on whether and when they completed the survey. Invitations ceased when a completed survey was recorded in REDCap. All forms of recruitment included the study flyer and a study letter, both of which stated that participants had three options to complete the survey: via mail, online, or over the phone. For example, the mailed letter and flyer had information on how to complete the survey by mail, online, or over the phone.

A small number of participants (*n* = 17, 1%) were mailed a study packet but did not have a valid mailing address (the mailed packet was returned as undeliverable) and, therefore, did not have the option of completing the survey by mail. A small minority of participants (*n* = 66, 3.9%) had neither a valid email address nor a valid cell phone number (both email and text message invitations were marked as undeliverable) and, therefore, did not have the option of completing the survey online. An additional sensitivity analysis was completed wherein those with a missing participation method were removed from the data. The removal of these records did not influence the interpretation of the results.

### Data collection

Survey data were collected between September 2022 and August 2023. Participants completed the survey either by mail, online, or by phone. Survey and recruitment materials were available in both English and Spanish. Patients whose primary language was listed as Spanish in the EMR were sent study materials in Spanish. A total of 1,679 cancer survivors participated in the survivor survey. Survivors from each of the 75 counties in Arkansas and 59 counties outside of Arkansas are represented in the study.

### Research questions

This study had two aims. First, we wanted to investigate whether rural or urban cancer survivors were more likely to participate in the survivorship survey. Because we did not have age information for invited patients, we could not assess the likelihood of survivorship survey participation by age. Second, we aimed to assess whether the mode of survivorship survey participation (mail, online, or phone) differed significantly among rural or urban cancer survivors and survivors of different ages.

### Data sources

**Figure 1.**
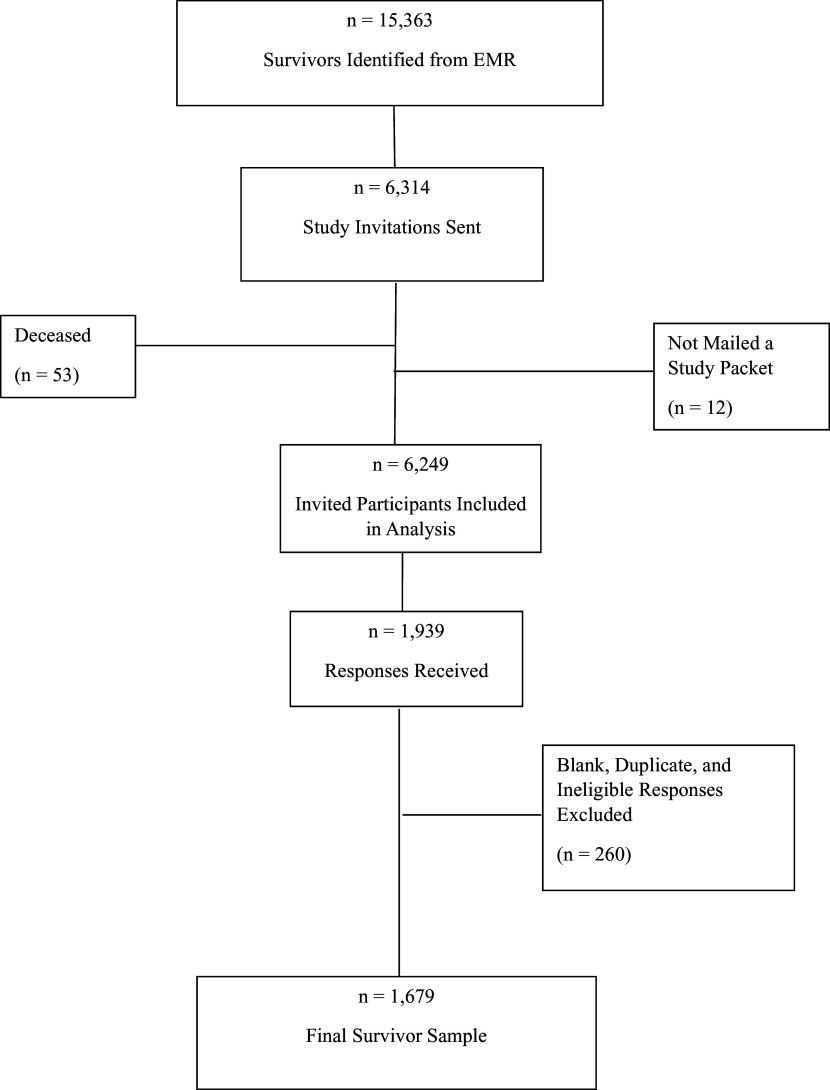
Study enrollment: cancer survivors.

We used data from all patients invited to participate in the survivorship survey and a dataset containing all valid participants in the survivorship survey. The study team identified 15,363 patients using EMR who met the inclusion criteria and were eligible for recruitment. The list of eligible patients was ordered randomly, and of those, a total of 6,314 patients were invited to participate during study recruitment. We removed 53 patients from the invited participants’ data whom the study team was informed were deceased during study recruitment (e.g., by a family member, postal service, etc.); these patients were not counted among the invited patients. We also removed 12 invited patients who did not have adequate home address information in the EMR and, therefore, were not mailed study packets, resulting in a total of 6,249 invited patients for this analysis. A total of 1,679 cancer survivors participated in the survivorship survey (see Figure 1).

### Variables

Study variables included rurality, age, and mode of survey participation. Patients’ home addresses were extracted from the EMR for the purpose of recruitment, and patients’ zip codes were used to categorize patients’ counties by rurality. Patients were coded as metropolitan (i.e., urban) or nonmetropolitan (i.e., rural) using Rural-Urban Continuum Codes (RUCC). RUCC scores range from 1 to 9, with 1 representing the most metropolitan and 9 representing the most nonmetropolitan. Scores 1–3 are classified as metropolitan and scores 4–9 as nonmetropolitan [17]. Survey participants were asked, “What is your age?” Participants’ mode of survey participation was recorded in REDCap as mail, phone, or online (via email or text message link).

### Outcomes

For the first research question, the outcome of interest was participation in the survivorship study, defined as completion of a valid survey either by mail, online, or phone. For the second research question, the primary outcome of interest was participation mode: either mail, online, or phone.

### Data analysis

After removing blank, ineligible, and duplicate surveys, the data were cleaned, coded, and analyzed using R (version 4.3.2). To assess participation rates, Pearson’s chi-square (χ2) test of independence was used to compare participation between rural and urban invitees and examine differences in participation methods among participants. To further investigate potential differences in participation mode, another χ2 test was conducted to explore the relationship between mode of participation (mail, online, or phone) and both participant rurality (urban or rural) and age group (age 44 or younger, 45–64, or 65 and older). To model the probability of each participation mode and estimate the effects of age and rurality, a multinomial log-linear model was fitted using the “nnet” R package.

An analysis of deviance table was used to assess how age, rurality, and their interaction influence participation mode choice. Similar to an ANOVA used with ordinary least squares regression, this approach evaluates each predictor’s contribution to explaining the variation in the outcome. The average effects of rurality and age (per year) on participation mode probability were estimated using the “marginaleffects” R package.

## Results

### Likelihood of study participation by rurality

Among invited patients, 2,646 (42.34%) resided in rural counties, and 3,603 (57.66%) resided in urban counties. A quarter (25.47%) of invited rural patients and 27.84% of invited urban patients participated in the survivorship study. We found the probability of participating in the survivorship study was approximately 1.09 times higher for cancer survivors in urban counties than cancer survivors in rural counties (χ^2^(1) = 4.31, *p* = 0.038).

### Mode of participation by rurality and age

A total of 1,677 participants had home address information and were categorized as having rural or urban county of residence. Forty percent (*n* = 674) of study participants resided in rural counties, and 1,003 (59.80%) resided in urban counties. There were significant differences between rural and urban survivors in the mode of survey participation (χ^2^(2) = 16.24, *p* < 0.001; Table 1).

**Table 1.** Differences in participation mode by rurality and age group

Characteristic	Mail, *n* = 985^1^	Online, *n* = 661^1^	Phone, *n* = 33^1^	*p*-value^2^
**Rurality**				<0.001
Rural	435 (44.16%)	230 (34.85%)	9 (28.13%)	
Urban	550 (55.84%)	430 (65.15%)	23 (71.88%)	
(Missing)	0	1	1	
**Age group**				<0.001
44 or younger	54 (5.56%)	144 (22.71%)	1 (3.13%)	
45–64 years old	308 (31.69%)	287 (45.27%)	14 (43.75%)	
65 and older	610 (62.76%)	203 (32.02%)	17 (53.13%)	
(Missing)	13	27	1	

1 *n* (%). ^2^Pearson’s chi-squared test.

A total of 1,638 participants had age data. A minority (12.14%) of participants were age 44 or younger, more than a third (37.18%) were age 45–64, and just over half (50.67%) were age 65 or older. There were significant differences by age group in the mode of survey participation (χ^2^(4) = 186.17, *p* < 0.001; Table 1).

**Figure 2.**
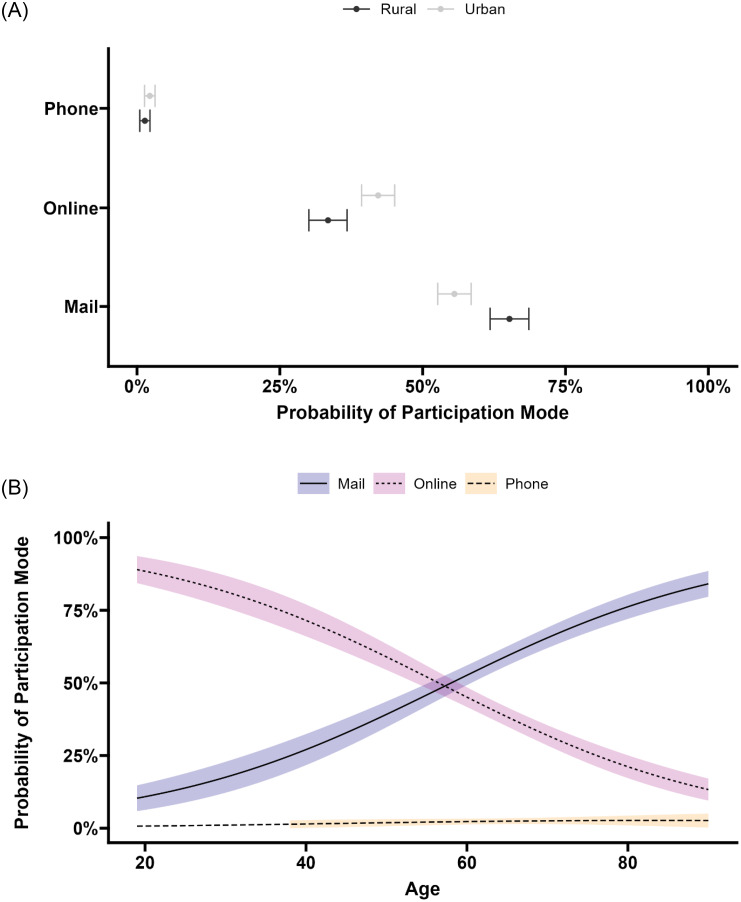
The predicted probabilities of each mode of participation showing the average effects of rurality (A) and age (B) with 95% confidence intervals derived from the predicted probabilities from the multinomial log-linear model.

To tease out the effect of both rurality and age, both were included in a multinomial model. Inspection of the analysis of deviance (analogous to an ANOVA) indicated an effect of both age (χ^2^(2) = 219.54, *p* < 0.001) and rurality (χ^2^(2) = 17.62, *p* < 0.001), but no interaction (χ^2^(2) = 1.07, *p* = 0.59). Therefore, the marginal effects were inspected to calculate the average effect of age and rurality (see Figure 2).

Rural survivors were significantly more likely to participate by mail (average difference [urban-rural] = −9.64%, *p* < 0.001; Figure 2A). Conversely, urban survivors were significantly more likely to participate online (average difference [urban-rural] = 8.77%, *p* < 0.001; Figure 2A). A minority of both rural and urban survivors participated by phone; the difference was not significant (average difference [urban-rural] = 0.87%, *p* = 0.19). In terms of relative difference, rural survivors were approximately 1.17 times more likely to participate by mail compared to urban survivors, while urban survivors were approximately 1.26 times more likely to participate online compared to rural survivors.

The average effect of age indicated an increase in participation by mail for each year of age by 1.16% (*p* < 0.001; Figure 2B). Meanwhile, we observed a reduction in the probability of participating online by approximately 1.20% per year (*p* < 0.001; Figure 2B). Although not significant (*p* = 0.23), participation by phone increased on average by 0.03% per year of age.

## Discussion

The goals of this study were to assess urban-rural differences in the likelihood of participation in a cancer survivorship survey and explore whether the mode of survey participation (mail, online, or phone) differed by urban-rural residence or age group among cancer survivors.

We found urban survivors were slightly more likely to participate in the cancer survivorship survey, with 28% of invited urban survivors and 25.5% of invited rural survivors completing surveys. The slightly higher participation rate among urban survivors likely reflects the fact that patients received treatment at UAMS, an academic medical center in the most urbanized area of the state. Overall, our findings align with extant literature showing rural residents are as interested in and willing to participate in health and cancer research as urban residents [11–13].

Rural cancer survivors were more likely than urban survivors to take part using a paper survey returned by mail, while urban survivors were more likely to participate using an email or text message link to REDCap and completing the survey online. Rural residents generally have less access to reliable, high-speed internet and cell phone coverage [18]. Rural residents also tend to be lower income and older and tend to have less education [17,19]. These are all potential barriers to using digital devices and the internet to participate in survivorship research.

Our study is one of the first to document that mailed surveys were used more by rural cancer survivors, while online survey completion was more common for urban survivors. Several cancer survivorship studies, including studies focused on rural cancer survivors, noted that mailed paper surveys were provided as an option or were the primary mode of data collection [8,14,20,21]. But no other studies, to our knowledge, have examined whether mailed surveys or other study completion modes are more likely to promote participation among rural populations. This is an important contribution to the literature, because rural residents remain underrepresented in cancer survivorship research. The National Cancer Institute has made increasing research with rural communities a priority [1]. The findings from this study could help researchers improve their efforts to engage rural community members in research, which is essential to gain the first-hand experiences of rural patients and survivors and generate community-informed knowledge.

Regarding age, we found the likelihood of participating by mail increased with age; conversely, the probability of participating online decreased as survivors’ age increased. While other studies have highlighted that older cancer survivors are less likely to participate in research [8] or may not be invited to participate in research at the same rates as younger survivors [15], no studies, to our knowledge, have assessed whether certain data collection modes may enhance research participation by older survivors. The findings of this study suggest non-digital methods, such as mailed study packets, are an important tool for engaging older cancer survivors in research studies.

Further, while a significant effect was not observed, the use of over-the-phone survey completion increased with survivors’ age, suggesting this may be a useful approach to promote participation among older cancer survivors, if study resources allow. Although only a small percentage of participants took part by phone, several of those participants expressed gratitude for being able to complete the survey by phone, noting limitations related to eyesight, ability to write, and ability to use the internet.

It is possible that the differences in the mode of participation between rural and urban cancer survivors may narrow in the coming years. As the younger generations age, both rural and urban cancer survivors may become more likely to participate in research via digital methods. However, any such narrowing of the gap in digital participation between rural and urban residents would depend on improvements in access to reliable, high-speed internet and cell phone coverage.

This study had several strengths, including the use of EMR to identify eligible cancer survivors and detailed tracking of recruitment and participation methods among invited survivors. The large and geographically diverse sample of cancer survivors enhances the generalizability of the findings. Additionally, we were able to compare the use of three distinct survey participation modes (mail, online, or phone) by rurality and age, an important contribution to the very limited research on effective data collection modes for underrepresented populations.

The study also had some limitations. We grouped those who participated via a text message link and those who participated via an email link together as “online participants.” It is possible that separating participants who used a text link and those who used an email link may have revealed important information on which digital participation modes were more likely by rurality and age. For example, it is possible that urban and younger survivors participated more via text message link than email link, while the opposite may have been true for rural and older survivors. In future work, we plan to analyze online research participation by text link vs. email link to tease apart which survivors are more likely to use these two distinct online participation modes. In addition, the study had age data for those who participated, but not for all of those who were invited. The absence of age data for invited survivors precluded evaluation of how age may have influenced the likelihood of participation in the survivorship survey.

Future work should engage both quantitative and qualitative methods to explore in-depth the drivers of survivorship research participation by rurality and age, as well as the barriers and facilitators of research participation for rural and older cancer survivors, who are often underrepresented in such work. Further, additional factors that may influence cancer survivorship research participation, such as cancer morbidity and social support, should be explored in future work.

Overall, the findings of this study highlight that providing a range of study participation methods for geographically- and age-diverse cancer survivors may be a key strategy to promote equitable access to cancer-related research. Our findings provide actionable information researchers can leverage to develop study designs that maximize participation among rural and older cancer survivors, two groups who remain underrepresented in cancer survivorship research. Such efforts are essential to meet the call of ASCO [16] and other leaders in the field to ensure equitable access to research for communities facing disproportionate cancer burden and higher risk of cancer disparities.
